# Neurological deterioration after posterior fossa decompression for adult syringomyelia: Proposal for a summarized treatment algorithm

**DOI:** 10.3389/fsurg.2022.968906

**Published:** 2022-09-15

**Authors:** Chenghua Yuan, Jian Guan, Yueqi Du, Zeyu Fang, Xinyu Wang, Qingyu Yao, Can Zhang, Zhenlei Liu, Kai Wang, Wanru Duan, Xingwen Wang, Zuowei Wang, Hao Wu, Fengzeng Jian

**Affiliations:** ^1^ Department of Neurosurgery, Xuanwu Hospital, Capital Medical University, Beijing, China,; ^2^ Spine Center, China International Neuroscience Institute (CHINA-INI), Beijing, China,; ^3^ Laboratory of Spinal Cord Injury and Functional Reconstruction, Xuanwu Hospital, Capital Medical University, Beijing, China; ^4^ Research Center of Spine and Spinal Cord, Beijing Institute of Brain Disorders, Capital Medical University, Beijing, China; ^5^ National Center for Neurological Disorders, Beijing, China

**Keywords:** syringomyelia, posterior fossa decompression, revision, pathology, c12 dislocation

## Abstract

**Background:**

Patients with syringomyelia who present with new neurological symptoms after posterior fossa decompression (PFD) are not uncommon. However, systematic reports on different pathologies are few in the literature.

**Objective:**

The purpose of this study was to summarize our experience for failed PFD.

**Methods:**

Between January 2015 and December 2019, 85 consecutive failed PFD patients were identified. The neurological courses were summarized with Klekamp J (KJ) or mJOA score system for all patients. Long-term results were summarized with Kaplan-Meier method.

**Results:**

Twenty-eight consecutive patients underwent FMDD (Foramen magnum and foramen of Magendie dredging) (Group I), extradural PFD and manipulation of tonsil was significantly associated with lower failure rates. Twenty patients underwent craniocervical fixation (Group II), nine underwent local spinal segment decompression (Group III), six underwent CSF diversion procedures, and one were treated for persistent pain by radiofrequency. Neuropathic pain was most significantly improved in Group I while swallowing improved in Group II within 1 year after the surgery. In the long term, late postoperative deterioration-free possibility in Group II was better than in Group I. All patients in Group III improved (*P* = 0.0088). Six cases of CSF diversion procedures were relieved in a short time. Pain in one patient persisted after PFD, and trial of radiofrequency failed.

**Conclusion:**

Not only does the recurrent cerebrospinal fluid flow obstruct the foramen magnum, but also spinal pathologies and craniocervical instabilities may occur. This study provides the largest summarized clinical experience that may assist surgeons with different therapeutic decisions for failed PFD.

## Introduction

Syrinx are most commonly associated with craniocervical anomalies, other causes include trauma, infections, degeneration, and tumors (1, 2). Syringomyelia may have multiple causes that require different treatment strategies. Posterior fossa decompression (PFD) remains the most commonly used treatment for patients with syringomyelia-Chiari complex; although important surgical details for such patients remain debatable (3). Persistent, recurrent, or worse syringomyelia after PFD occurs in 10%–50% of cases (4, 5). Repeat PFD has been the first-line procedure for failed PFD. Atlantoaxial fusion and other possible spinal pathology treatments or shunting procedures are other treatment options (6–9).

To date, few reports have been published on the indications and treatment choices for persistent or recurrent syrinx, or worsening neurologic symptoms after PFD (5, 7, 8). Moreover, existing articles do not systematically summarize the treatment processes for syringomyelia based on the different underlying pathologies.

The aim of this study was to review our experience for clinically treating patients with syringomyelia for whom PFD had previously failed.

## Methods

The study was reviewed and approved by the local ethics committee with waiver of informed consent from patients given its retrospective nature.

Between January 2015 and December 2019, 85 consecutive patients with failed PFD for syringomyelia were treated at our institution. For most patients, the first surgery had been conducted at other institutions. The indications for first surgery included: progressive neurological symptoms or syringomyelia.

**Figure 1:**
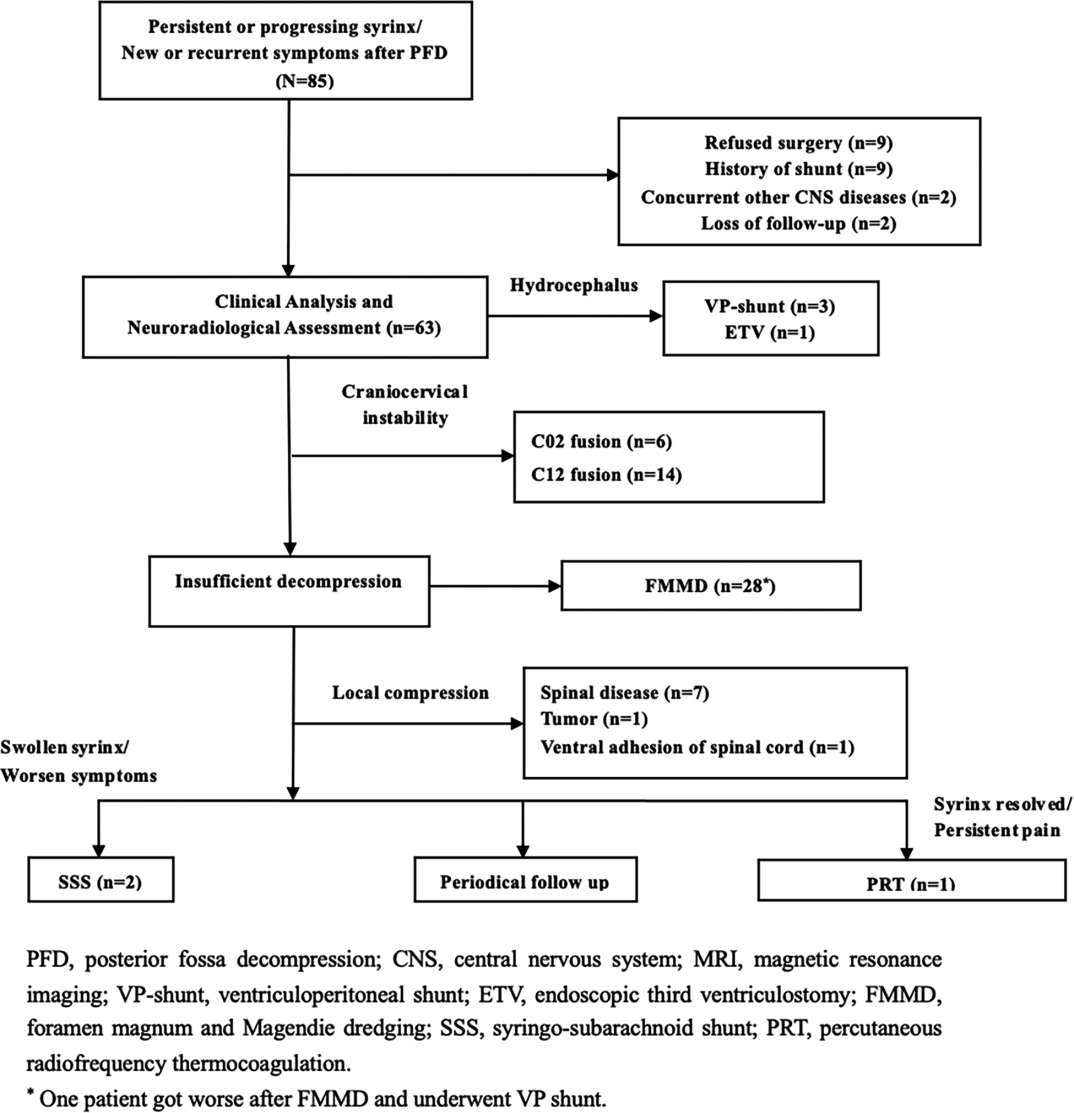
The treatment algorithm categorization and surgical strategy of 85 consecutive failed PFD patients between 2015 and 2019.

The indications for secondary surgery included: (1) new, recurrent, or progressive neurological symptoms (independent of syrinx size), (2) large or swollen syringomyelia with obvious spinal cord compression (10), (3) progression of syringomyelia over time despite no or few symptoms, or other obvious pathology. Patients were excluded if they had a history of shunt placement, infection, multiple decompression, or incomplete follow-up data. The patients were categorized according to the flow chart shown in Figure 1.

KJ scores (11) and mJOA were used to evaluate the clinical manifestations of Group I, II and III before and after surgery. Long-term results were summarized with Kaplan-Meier statistics.

### Clinical categorization

Patients were divided into three groups according to their neurological symptoms, radiological findings; and postoperative outcomes of the first PFD.

#### Group I

**Figure 2:**
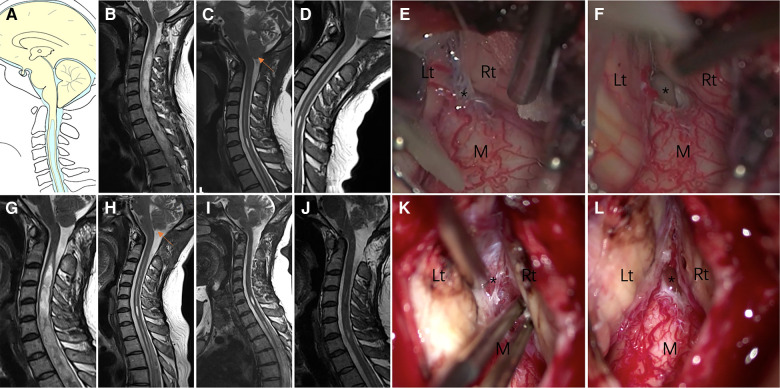
Case presentation of Group I, a 31-year-old woman presented with upper extremity paresthesia. (**A**) Schematic drawings of the foramen magnum region, sagittal view. Midsagittal T2-weighted MRI scans of the cranio-cervical before the initial craniocervical decompression surgery (**B**), 3 (**C**) months after the initial surgery, and 6 (**D**) months after the initial surgery are shown. (**E,F**) An obvious veil obstructing the foramen of Magendie (asterisk). (**G**) 1 year after the initial surgery. Postoperative MRI after 3 months (**H**), 6 months (**I**) and 1 year (**J**) of revision surgery showing good decompression of the posterior fossa. (**K,L**) Veil obstructing the foramen of Magendie was removed and tonsil was coagulated. Lt, left tonsil; Rt, right tonsil; M, medulla oblongata.

Patients were categorized as Group I if insufficient decompression (Figure 2) was achieved. Clinical symptoms were attributed to the brainstem, cerebellum, and lower cranial nerves, or if the upper segment of the syrinx was at the foramen magnum level (10, 12). “Good decompression” was defined as effective spinal cord decompression where the original obstruction to CSF flow was caused by the ectopic cerebellar tonsil, as observed on MR images, and reappearance of the subarachnoid cisterns (Figure 2) of the posterior fossa between the brainstem and cerebellar tonsil.

#### Group II

Patients were placed in this group if their dynamic radiographic and reconstructive CT images indicated cranio-cervical instability, and if their clinical symptoms were related to the brainstem, cerebellum, and lower cranial nerves.

#### Group III

If preoperative MR images demonstrated obvious local spinal pathology, the patient was categorized in Group III. For these patients, direct local segment decompression, removal of the lesion, and arachnoid lysis were the surgical options considered.

### Surgical treatments

#### Foramen magnum and foramen of magendie dredging (FMMD)

Our detailed surgical techniques for FMMD in patients with failed PFD have been published previously (8, 13). In brief, all patients in Group I underwent secondary suboccipital decompression combined with careful intradural exploration (8). Different techniques were adopted based on herniation status of the cerebellar tonsils, i.e., low-power bipolar electrocoagulation for mild herniation or medialized hypertrophy and subpial resection for obvious herniation. Electrocoagulation was used to seal the pial opening to ensure hemostasis. Maintaining the integrity of the pia mater is also essential for preventing potential adhesion, scarring, or recurrence. For other pathologies like inter-tonsillar arachnoid adhesions, tonsil to medulla arachnoid adhesions, tonsil to dura mater arachnoid adhesions, and arachnoid veil, micro scissors are used to separate the adhesions (8).

#### Direct posterior reduction and fixation

Patients in Group II underwent direct posterior reduction and fixation. The main challenge of this revision surgery was the presence of an occipital defect; the initial operation had destroyed the posterior nuchal attachment and the neck muscles, which caused subsidence at the cranial-cervical junction. In the second surgery, we distracted the facet joints, restored the lost perpendicular height, and then reduced the horizontal translation. The following techniques for posterior fixation were used:

(a) C1 lateral mass screw and C2 pedicle screw fixation (14).
(b) Occiput to C2 fixation with C2 pedicle screws (15).

#### Local spinal decompression

The techniques for cervical spondylosis compression include anterior or posterior decompression (16). For lumbar compression fracture, some authors have suggested an anatomy-based comprehensive classification of spinal osteotomies (17). Most astrocytoma was biopsied; however, tumor resection using an inside-out technique can be adopted (18). As for patient who develop ventral cyst of the spinal cord after PFDD, we tried cyst incision and drainage.

#### CSF diversion procedures

The relationship between CM and hydrocephalus is interesting. There is limited evidence for surgical management of CM associated with hydrocephalus (19). SSS (syringo-subarachnoid shunt) should be the last procedure to be considered because of its higher failure rate (20).

#### Pain persisted

Persistent neurogenic pain after decompression is not uncommon. SCS (Spinal cord stimulation) is an effective method for the treatment of pain (21, 22). Pulsed radio frequency (PRF), which is a type of undifferentiated pain treatment method, uses radio frequency current to produce high voltage pulse in a short time; this blocks nociceptive afferent pathways (23). However, PRF was failed.

### Postoperative assessment

Follow-up information was obtained during outpatient visits or by telephone interviews. Treatment success was defined as sustained improvement of preoperative symptoms or stabilization of previously progressing symptoms. Treatment failure was defined as postoperative neurological deterioration. Patients were assessed at postoperative, 3 months and 12 months postoperatively for neurological recovery using the KJ or mJOA scores (24). Long-term results were summarized with Kaplan-Meier statistics in three groups. Patients also underwent postoperative radiography, reconstructive CT, and MRI to determine the volume of syrinx, decompression, bone graft status, and reduction state.

### Statistical analysis

The baseline data of the Group I and II of patients were summarized using descriptive statistics: Student's *t* test, the Mann–Whitney *U* test, the chi-square test or Fisher's exact test (SPSS 25.0, Chicago, IL). The KM method were employed to compare the deterioration-free survival between the three groups (R version 4.0.2).

## Results

Group I had 28 patients (8 men and 20 women with a mean age of 40.5 ± 12.5). Group II had 20 patients who presented typically with swallowing problems, and compared to Group I, had a longer interval between the first and second surgeries (39 ± 33 vs. 60 ± 24 months). Hypesthesia, weakness of the limb(s), and Neuropathic pain were the top three main complaints. The clinical presentations and underlying etiologies are summarized in Table 1.

**Table 1:** Clinical or radiological data and management strategies of Group I and II patients (*n* = 48).

Variable	Group I (*n* = 28)	Group II (*n* = 20)
**Clinical data**		
Age at second admission (years)	47.6 ± 11.3	42.6 ± 9.0
Sex (male)	8 (40.0%)	4 (20.0%)
Age at initial diagnosis	42.0 ± 11.9*	32.1 ± 11.3*
**First preoperative symptom duration (months)**		
<6	9 (32.1%)	7 (35%)
6–2 years	8 (28.6%)	9 (45%)
>2 years	11 (39.3%)	4 (20%)
Surgery interval (months)	59.1 ± 59.2*	111.7 ± 86.1*
**Symptoms**		
Occipital pain	10 (35.7%)	3 (10.7%)
Neuropathic pain	18 (64.3%)	7 (25.0%)
Dysesthesia	22 (78.6%)	15 (75.0%)
Hypesthesia	23 (82.1%)	17 (85.0%)
Motor Power	18 (64.3%)	19 (67.9%)
Gait	16 (57.1%)	16 (57.1%)
Sphincteric dysfunction	3 (10.7%)	2 (7.1%)
Swallowing Function	7 (25.0%)	9 (32.1%)
**Radiological data**		
Atlantoaxial dislocation	0*	17 (85.0%)*
Occipitalization of atlas	4 (14.3%)*	17 (85.0%)*
Basilar invagination	6 (21.4%)*	17 (85.0%)*
Klippel Feil	0*	10 (50.0%)*
Platybasia	7 (26.9%)	9 (32.1%)
CXA	147 ± 10.8*	138 ± 7.4*
Hydrocephalus	1 (3.6%)	0
Scoliosis	5 (17.9%)	2 (10.0%)
Syringomyelia	26 (92.9%)	17 (85.0%)
Diameter (cm)	0.7 ± 0.2	0.6 ± 0.3
**Location**		
Cervical	3 (11.5%)	5 (29.4%)
Cervicothoracic	23 (88.5%)	12 (70.6%)
Holocord	0	0
**First management**		
Extradural	13 (46.4%)	14 (70.0%)
Intradural	15 (53.6%)	6 (30.0%)
**Intradural findings**		
Veil over FM	11 (39.3%)	NA
Hypertrophic tonsils	19 (67.9%)	NA
PICA adherence	4 (14.3%)	NA
Pseudomeningocele formation	1 (3.6%)	1 (5.0%)
Intertonsillar adhesions	2 (7.1%)	NA
**Syrinx outcome**		
Resolved	18 (69.2%)	8 (47.1%)
Unchanged	7 (26.9%)	9 (52.9%)
Worsen	1 (3.9%)	0
**Complication**		
Aseptic meningitis	5 (17.9%)	1 (5.0%)
CSF fistula	2 (7.1%)	0
Hydrocephalus	1** (3.6%)	0
Swallowing dys	1** (3.6%)	0
Hemorrhage	0	1 (5.0%)
Wound infection	1** (3.6%)	0
Urinary tract infection	1** (3.6%)	0
Pneumonia	1** (3.6%)	0
**Length of follow-up** (months)	23.6 ± 10.9	29.2 ± 15.9

FM, foramen of magendie; PICA, posterior inferior cerebellar artery; CXA, clivoaxial angle.

*Significant difference (*p* < 0.05) between groups.

**One patient had these symptoms at the same time.

All 28 patients in Group I with insufficient decompression were treated with FMDD. Intraoperatively, management of intradural factors included addressing adhesion between the cerebellar tonsils and dura mater arachnoid, adhesion of the PICA or its branches, descent of the cerebellar tonsil and medulla (Figure 2). Most (78.6%) of patients either clinically improved or remained stable. Five patients were diagnosed with aseptic meningitis, which was treated successfully by intermittent lumbar puncture. Two patients developed a CSF fistula after the operation. In one patient, dysphagia was not relieved and fever persisted after decompression. The symptoms were controlled after VP shunt. However, the patient died 3 years later.

**Figure 3:**
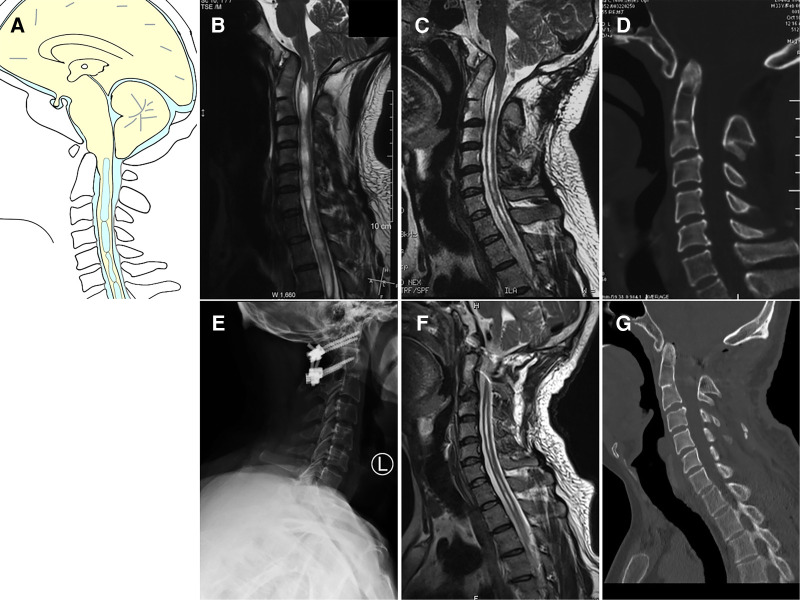
Case presentation of Group II, a 33-year-old man with dizzineess who received initial suboccipital decompression without fusion 6 months ago. The symptoms had worsened gradually. (**A**) Schematic drawings of the foramen magnum, sagittal view. (**B**) Preoperative MRI (sagittal view) showed ventral compression of the cervicomedullary junction, Chiari malformation and syringomyelia. (**C**) Preoperative MRI scan showed syringomyelia was reduced. (**D**) Preoperative CT scan showed basilar invagination, atlantoaxial dislocation and occipital bone defect. (**E**) Postoperative X showed C1 lateral mass screw and C2 pedicle screw fixation. (**F**) Postoperative MRI showed good decompression of the cervicomedullary junction. (**G**) Postoperative CT indicated complete reduction of both basilar invagination and atlantoaxial dislocation.

The twenty patients in Group II with craniocervical instability were treated with either posterior C1–C2 fixation (*n* = 14) (Figure 3) or occipitocervical fixation (*n* = 6). All patients achieved complete decompression after posterior reduction and fixation. Clinical improvement was achieved in nearly all patients. Only two patients experienced any intra- hemorrhage or postoperative fever complications.

Regarding symptom improvement after surgery (Table 2), for patients in Groups I and II, substantial symptom improvements were noted in neuropathic pain, dysphagia, whereas the remainder of symptoms only improved marginally. Radiological improvements were noted in 69.2% or 47.1%, individually. The overall complication rate for Group I and II was 28.6% and 10.0%, respectively.

**Table 2:** Neurological course of Group I and II patients after the revision surgery (*n* = 48)*.

Symptom	Group I (*n* = 28)	Group II (*n* = 20)	All
**Occipital pain**			
Preop	4.4 ± 0.8	4.9 ± 0.5	4.6 ± 0.7
Postop	4.5 ± 0.7	4.9 ± 0.5	4.7 ± 0.7
3 months	4.7 ± 0.5	4.9 ± 0.5	4.7 ± 0.5
1 year	4.7 ± 0.5	4.9 ± 0.2	4.8 ± 0.4
**Neuropathic pain**			
Preop	3.6 ± 1.0	4.6 ± 0.7	4.0 ± 1.0
Postop	4.0 ± 0.9	4.7 ± 0.7	4.3 ± 0.9
3 months	4.1 ± 0.8	4.7 ± 0.7	4.4 ± 0.8
1 year	4.1 ± 0.9	4.8 ± 0.5	4.4 ± 0.8
**Dysesthesia**			
Preop	3.3 ± 1.1	3.3 ± 1.2	3.3 ± 1.1
Postop	3.4 ± 1.1	3.6 ± 1.0	3.4 ± 1.1
3 months	3.4 ± 1.1	3.7 ± 1.2	3.5 ± 1.1
1 year	3.3 ± 1.1	3.9 ± 1.1	3.6 ± 1.1
**Hypesthesia**			
Preop	2.9 ± 1.1	2.9 ± 1.0	2.9 ± 1.1
Postop	3.0 ± 1.1	2.9 ± 1.0	3.0 ± 1.1
3 months	3.0 ± 1.1	3.0 ± 1.1	3.0 ± 1.1
1 year	3.0 ± 1.1	3.1 ± 1.0	3.1 ± 1.1
**Motor weakness**			
Preop	4.1 ± 0.8	4.0 ± 0.5	4.0 ± 0.7
Postop	4.2 ± 0.7	4.2 ± 0.6	4.2 ± 0.7
3 months	4.3 ± 0.7	4.4 ± 0.6	4.3 ± 0.6
1 year	4.3 ± 0.7	4.4 ± 0.6	4.3 ± 0.7
**Gait ataxia**			
Preop	3.9 ± 1.2	3.0 ± 1.1	3.5 ± 1.2
Postop	4.0 ± 1.0	3.3 ± 1.0	3.7 ± 1.1
3 months	4.1 ± 1.1	3.5 ± 1.0	3.8 ± 1.1
1 year	4.1 ± 1.0	3.7 ± 0.8	3.9 ± 0.9
**Bladder function**			
Preop	4.8 ± 0.6	4.8 ± 0.6	4.8 ± 0.6
Postop	4.8 ± 0.6	4.8 ± 0.6	4.8 ± 0.6
3 months	4.9 ± 0.4	4.8 ± 0.6	4.9 ± 0.5
1 year	4.9 ± 0.4	4.9 ± 0.5	4.9 ± 0.4
**Swallowing**			
Preop	4.5 ± 0.8	4.0 ± 1.2	4.3 ± 1.0
Postop	4.6 ± 0.7	4.6 ± 0.8	4.6 ± 0.8
3 months	4.7 ± 0.6	4.7 ± 0.7	4.7 ± 0.6
1 year	4.7 ± 0.7	4.7 ± 0.7	4.7 ± 0.7
**Overall**			
Better	71.4%	90%	79.2%
Unchanged	7.2%	5%	6.3%
Worsen	21.4%	5%	14.6%

*Unless otherwise specified, all values are expressed as the mean ± SD.

**Figure 4:**
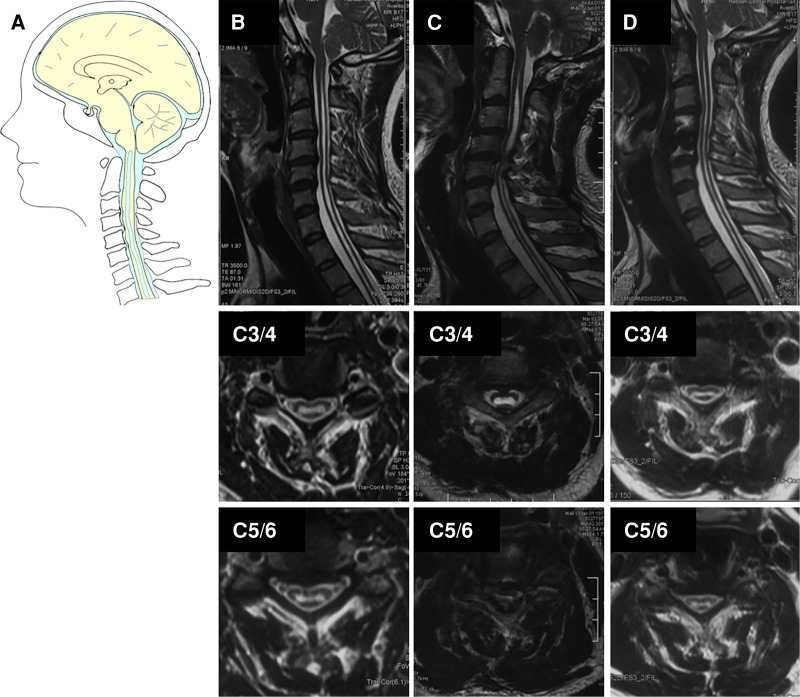
Case presentation of **cervical disc herniation** in Group III, (**A**) schematic drawings of the foramen magnum, sagittal view. **Sagittal (upper), axial (middle, C3/4), and axial (down, C5/6)**. (**B**) Preoperative MRI showed the Chiari I malformation and syrinx. (**C**) Postoperative MRI after 4 years showed obvious C4/5-disc herniation and enlarged cervical syrinx above C4/5 level (red arrow). (**D**) A postoperative MRI after ACDF showed the cervical spinal canal was completely decompressed and the syrinx above C4/5 was partially resolved and lower extremity inflexibility improved.

**Figure 5:**
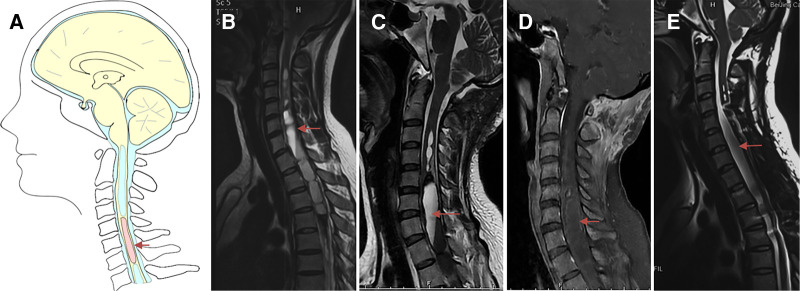
Case presentation of **spinal cord tumor** in Group III, a 28-year-old woman with progressive weakness and numbness of both lower limbs who received failed suboccipital decompression in local hospital 1.5 months ago. (**A**) Schematic drawings of the cervical spine, sagittal view. (**B**) Preoperative sagittal T2-weighted MRI showed large syringomyelia. (**C,D**) Postoperative enhanced MRI showed partial bone defects of posterior inferior border of occipital bone, spinal cord thickening with abnormal signal at the level of C5-T6, central canal dilation at C3-5 and T7-9, previous intramedullary hemorrhage at T10-11 level. (**E**) Postoperative MRI after 6 months showed obvious reduction of syringomyelia. Under electrophysiological monitoring, the tumor was completely removed, and the postoperative pathological results were astrocytoma, WHO grade I. Arrow: spinal cord tumor.

**Figure 6:**
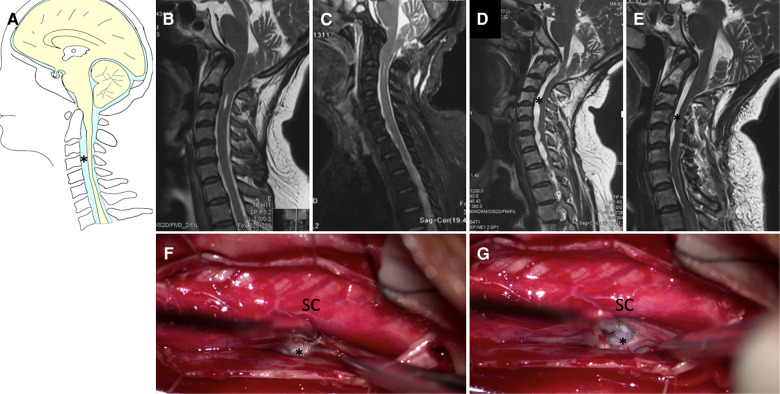
Case presentation of **ventral cyst** of spinal cord in Group III, (**A**) schematic drawings of the cervical spine, sagittal view. (**B**) Preoperative sagittal T2-weighted MRI showed small syringomyelia. (**C**) 2 weeks, (**D**) 3 months later after the first surgery MRI showed partial bone defects of posterior inferior border of occipital bone, ventral cyst of spinal cord. (**E**) Postoperative MRI after second surgery showed obvious reduction of cyst. Under electrophysiological monitoring, the cyst was incised and drained (**F,G**). SC, spinal cord. Asterisk: Ventral cyst.

**Figure 7:**
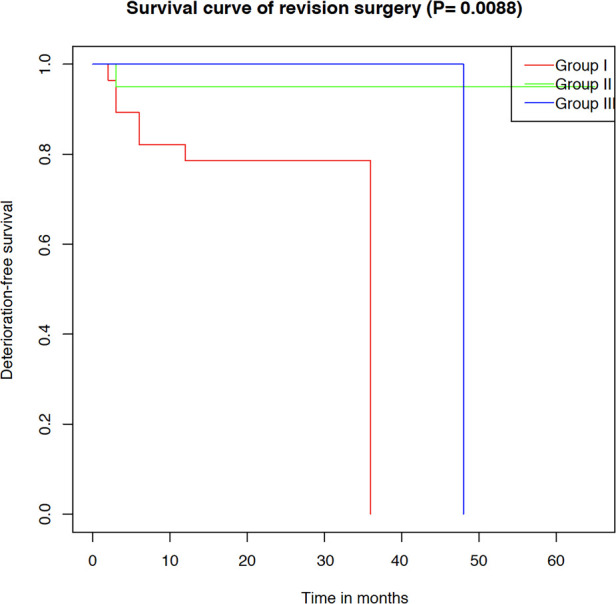
Kaplan–Meier analysis illustrating deterioration-free survival in different groups.

As for the six degenerative spinal disease patients in Group III (Table 3), clinical improvement or stable was achieved in all patients. One patient with cervical disc herniation was treated with anterior cervical decompression and fusion at the C4/5 level (Figure 4). Postoperative MR images revealed favorable decompression. Another patient with lumbar compression fracture was treated with L2 vertebral column resection and cage implantation (25). The third patient had tumor, which was misdiagnosed as CM and syringomyelia, and was treated by PFD. This patient was transferred to our hospital, where a total tumor resection was performed under electrophysiological monitoring (Figure 5). The pathological findings of the biopsy specimens indicated astrocytoma. The last patient had ventral cyst of spinal cord after PFDD. This patient was transferred to our hospital, where a cyst incision and drainage was performed under electrophysiological monitoring (Figure 6). In terms of long-term prognosis, the possibility of deterioration-free possibility in Group II was better than that in Group I, and all patients got improved in Group III [*P* = 0.0088, (Figure 7)].

**Table 3:** Clinical or radiological data and management strategies of other patients except Group I and II (*n* = 16).

No	Age/sex	Initial symptoms; JOA	Preop Syrinx	Initial surgery	Symptom outcome	Postop syrinx	Interval (years)	Symptoms; JOA	Second surgery	Final outcome; JOA	Follow-up (years)
1	54/F	Diz, Gi, Bi Ue M, Ue and T S, U, 10	C2-T	Intradural C45 lam	Improved for 15 years	Reduced	16	Gi, Bi Ue M, Ue and T S, U; 10	C56 ACDF	Improved, but Ue M worsen 4 years later; 11	5
2	61/F	Lt Ue S and M, 15	C2-T1	Intradural	Worsen	Reduced	1	Le S and M, Rt Ue S; 12	C56 ACDF	Improved; 13	2
3	48/F	Diz, Lt Ue and T S and *P*, Lt Hl; 14	T3-5	Extradural	Stable	Slight Reduced	3 months	Diz, Lt Ue and T S and *P*, Lt Hl; 14	C56 ACDF	Improved; 15	2
4	50/M	Sw, Bre, Rt S and M, Rt Ue or T *P*; 12	C0-L2	Intradural	Improved for 6 months	Reduced	1	Sw, Rt S and M, Rt Ue and T *P*; 9	L2 cage implant	Improved; 11	2
5	47/M	Rt Ue S and M; 15	C2-T	Intradural	Improved for 3.5 years	Reduced	4	Bi Ue S and M; 13	C45 ACDF	Improved; 15	2
6	61/F	Ataxia, Diz; 14	None	Extradural	Improved	–	8	Diz, Cer *P*; 14	C56 ACDF	Improved; 15	2
7	50/M	Lt Ue S and M, 13	C2-T6	Extradural	Improved for 3 years	Stable	10	Bi Ue S and M; 11	C56 ACDF	Improved; 13	1
8	27/F	Rt Le S and M, 14	C3-T6	Extradural	Worsen	Stable	2 months	Bi Le S and M, T S; 13	Tumor removed	Improved; 15	1
9	51/F	Diz, Gi; 15	C3	Intradural	Worsen	Stable	1	T S, Bi Le S and M, U; 10	Cyst incision	Improved, 12	1
10	51/F	Ha, Gi, Rt Ue S, *P*; 12	C2-T	Intradural	Improved	Stable	2	Rt Ue S, *P*; 14	Rad	Unchanged; 14	3
11	51/F	Diz, Gi, 15	None	Extradural	Improved for 5 years	–	6	Diz, GI; 14	VP-Shunt	Improved; 15	1
12	47/F	Ha, Cer *P*, Swa, Gi, Lt eye *P*; 14	None	Extradural	Improved for 1 mo	–	4 months	Ha, Cer *P*, Swa, Gi, Lt eye *P*, Diz; 14	FMDD, VP-Shunt	Died after 3 years	3
13	36/M	Lt Ue S; 16	C2-T	Intradural	Unchanged	Reduced	12	Lt Ue S, 16, 13	VP-Shunt, FMDD	Improved; 14	13, 3
14	14/F	Ha, Gi; 13	None	Intradural	Improved for 1 year	-	2	Ha, Gi; 13	ETV	Improved; 15	4
15	71/F	Ha, Rt Ue and T S, *P* and M; 9	C2-T	Intradural	Worsen	Stable	3	Bi Ue and T S, *P* and M; 7	SSS	Improved; 9	2
16	38/F	Ha, Diz, Ba *P* Lt Ue S, M; 11	C2-T	Intradural	Unchanged	Stable	2	Lt Ue S, M; Ba *P*; 11	SSS	Improved; 12	5

Diz, dizziness; Gi, gait instability; Bi, bilateral; Ue, upper extremity; Le, lower extremity; M, motor deficit; T, trunk; S, sensory abnormalities; U, urinary incontinence; Hl, hearing loss; Sw, swallowing abnormalities; Bre, breathing abnormalities; Cer, cervical; HA, headache; Lt, left; Rt, right; *P*, pain; Ba, back; Rad, radiofrequency; ETV, endoscopic third ventriculostomy.

For six patients with CSF shunt, two patients with syrinx-subarachnoid shunt were relieved or stable, while for the other one patient with ETV was relieved. The other three patients with ventriculoperitoneal shunt were all relieved in a short period. One patient died 3 years later, and one patient deteriorated 10 years later and underwent decompression again.

For the patient with persistent pain after decompression, the patient's pain lasted until two years after operation. Trial of cervical nerve root radiofrequency treatment (twice) resulted, in relief of postoperative pain for a short time; the pain recurred after about two months.

## Discussion

Conditions such as CMI, trauma, arachnoiditis, spinal degenerative diseases, and epidural and extramedullary tumors often lead to occlusion of the subarachnoid space followed by disturbances in CSF flow resulting in syringomyelia (25–27). However, for intramedullary tumors or vascular lesions, transudation and tumor cell secretion are responsible for syrinx progression (28). The causes of treatment failure in patients with syringomyelia have been investigated previously (5, 7, 8, 29). However, there is a lack of systematic summary of large number of cases.

### Preoperative assessment

For each case of symptomatic syringomyelia, irrespective of previous surgery status, it is important to exclude hydrocephalus and brain tumor as underlying causes. This is followed by detailed evaluations to determine the symptomatology both before and after the initial decompression surgery, as well as symptom changes over time. In this revision study, we noted that the incidence of occipital headache was low, which was related with the effect of first bone decompression. Clinical symptoms associated with the brainstem, cerebellum, or lower cranial nerves, including swallowing dysfunctions and ataxia, usually improved in patients in Groups II. However, for non-functional symptoms, such as neuropathic pain, the clinical improvements were obvious in these Groups I patients. Indeed, hypesthesia was a common complaint of patients included in this study. This type of hypesthesia often remained after decompression. Therefore, it is necessary to tell a patient before the operation that their hypesthesia may not improve even after successful decompression except slight paresthesia. The medical history of some patients revealed an interval of clinical stability after the first PFD, with later re-emergence of swallowing dysfunctions. These symptoms suggest that the deterioration is related to the foramen magnum (8).

In our cohort, the foramen magnum area was first evaluated by comparing the preoperative and postoperative MRI findings for the first failed PFD, including the size of the cisterna magna and extent of decompression of cerebellar ptosis, pseudo meningocele formation, anterior compression of the odontoid, and/or craniocervical instability. Several researchers (8, 10, 30) have suggested that the upper segment of the syrinx near the foramen of magendie indicates underlying pathological changes in the foramen magnum area, which can be removed at the craniocervical level to restore free CSF flow.

Another important factor in the assessment of patients in whom PFD initially failed is the course of a syrinx after surgery. If a syrinx has returned, the reason for this needs to be investigated and the syrinx may need to be treated at the original area. Persistent syrinx may also be due to insufficient decompression or other underlying pathology. In our cohort, operative records from other institutions rarely contained video of the first decompression. If syrinx enlargement was above the compression level, it was likely that the new symptoms were related to local compression. If all of these factors were excluded based on MR images, whole-spine or enhanced MRI was performed to evaluate the patient for other pathologies. The main objective when performing a secondary decompression in patients for whom the initial PFD failed is clinical stabilization of the progressive clinical course. For the majority of the patients, periodical follow-up was recommended because their clinical symptoms were stable or only changed slightly, and because their syrinx had reduced in size (26).

### Group I

While it is difficult to anticipate intraoperative findings until the dura is opened, accessing information related to the previous decompression surgery, such as the extent of bony resection or patency of the foramen of Magendie, is important when performing a secondary surgery, as it may guide the surgeon's decisions regarding whether to explore the subarachnoid space and whether to resect or coagulate the tonsils.

The secondary decompression may assist in identifying the cause of the first failed decompression. Intradural manipulation may cause underlying adhesions, but extradural decompression is often not sufficient to reestablish free CSF flow. The importance of opening the foramen of Magendie during PFD has been reported (8, 29, 31–33).

### Group II

Indeed, preoperative instability can be easily overlooked. If dynamic radiographs or CT images show excessive activity at the craniocervical junction or upper cervical spine during flexion and extension, it is necessary to consider decompression combined with appropriate fusion. Some patients with craniocervical instability may not be identified because of unstable compensation before the operation, but this instability can become aggravated after the operation due to separation of the cervical muscles from the occipital region.

Surgical revision of craniocervical instability is difficult, especially for those patients who have previously undergone PFD. The large occipital defect and the loss of anatomical structure on the scar plane causes this dilemma.

In Group II, patients whose first surgery involved PFD without fusion had aggravated postoperative symptoms. Instability and disruption of the posterior tension band may have led to the failure of the first operation. In this case, the strategy we used during the secondary surgery was first to distract the facet joints, and then to restore the lost perpendicular height and reduce the level shift. In Group II, some patients were instrumented with C1 lateral mass screw and C2 pedicle screw fixation, and the other patients received occiput to C2 fixation with C2 pedicle screws (14, 15, 34). Detailed surgical strategies based on anatomy and preoperative planning are important to avoid hypoglossal nerve root and vertebral artery horizontal segment.

### Group III and other considerations

Extramedullary lesions that produce chronic spinal cord compression are a factor in the formation of syrinx. Compressive pathogenic factors include cervical disc herniation, basal impression, post-traumatic compression fracture, and arachnoid cyst (28, 35). Although cervical spondylosis is a common condition, it is rarely associated with syringomyelia (16, 36, 37). Syringomyelia is rarely related to spinal cord injuries (25, 38). The detailed medical history of a patient in Group III with this presentation has been summarized and published previously (25).

The most common tumor types associated with syrinx are ependymomas and hemangioblastomas, but astrocytoma are rare (28, 39, 40). Spinal arachnoiditis cyst is a rare complication of subarachnoid hemorrhage (41).

Some authors suggest shunting of the syrinx as an alternative choice to failed surgery (6). However, the surgeons at our institute avoid shunting unless the underlying pathology related to the syrinx cannot be found by existing methods. There is no high-level evidence for surgical management of syringomyelia associated with hydrocephalus (42). Studies analyzing the possible reasons for developing hydrocephalus after PFD are also limited.

It is not uncommon that syringomyelia related neuropathic pain does not relieve continuously after decompression. Common methods of pain relief include analgesics, radiofrequency and spinal cord stimulation; (21–23) in our patient use of the PRF were unsuccessful.

### Other considerations and limitations

Although this is a small cases series, the descriptions of the revision surgeries for failed PFD-related syringomyelia, including the various methods and techniques used, may help other surgeons plan similar revision surgeries. It is also important for surgeons to understand the key underlying pathology that led to failure of the initial surgery. Etiologies for the failed initial operation are multifactorial.

This study had some limitations, particularly its retrospective design, which may have introduced bias related to the way surgeons operate at our center. Furthermore, most of the patients included in this study were referred to us after undergoing the first PFD at other institutions. Therefore, the factual incidence of recurrent symptoms or syringomyelia in the group is difficult to determine.

## Conclusions

The management of patients with PFD failure depends on their underlying pathology. The different surgical techniques for patients with syringomyelia should be carefully reevaluated to reduce failure rates.

## Data Availability

The original contributions presented in the study are included in the article/Supplementary Material, further inquiries can be directed to the corresponding author/s.
